# Novel Bioactive Titanate Layers Formed on Ti Metal and Its Alloys by Chemical Treatments

**DOI:** 10.3390/ma3010048

**Published:** 2009-12-25

**Authors:** Tadashi Kokubo, Seiji Yamaguchi

**Affiliations:** Department of Biomedical Sciences, College of Life and Health Sciences, Chubu University, 1200 Matsumoto-cho, Kasugai, Aichi, 487-8501, Japan; E-Mail: sy-esi@isc.chubu.ac.jp (S.Y.)

**Keywords:** bioactive, titanate, osteoinduction, osteoconduction, Ti metal, Ti-based alloy, artificial joint, spinal fusion device, apatite

## Abstract

Sodium titanate formed on Ti metal by NaOH and heat treatments induces apatite formation on its surface in a body environment and bonds to living bone. These treatments have been applied to porous Ti metal in artificial hip joints, and have been used clinically in Japan since 2007. Calcium titanate formed on Ti-15Zr-4Nb-4Ta alloy by NaOH, CaCl_2_, heat, and water treatments induces apatite formation on its surface in a body environment. Titanium oxide formed on porous Ti metal by NaOH, HCl, and heat treatments exhibits osteoinductivity as well as osteoconductivity. This is now under clinical tests for application to a spinal fusion device.

## 1. Introduction

Various types of bone-bonding, bioactive ceramics have been developed and have been used clinically as bone substitutes [1]. However, all of these exhibit poor fracture toughness and hence cannot be used under load-bearing conditions, such as those experienced by artificial joints and dental implants. Titanium (Ti) metal and its alloys coated with hydroxyapatite are sometimes used for such purposes. However, the coated layer is not stable in the living body for long periods, and so the development of bioactive materials with a high fracture toughness is desired.

The present authors found early on that a titania gel prepared using a sol-gel method forms a bone-like apatite layer on its surface in a simulated body fluid (SBF) with ion concentrations almost equal to those of human blood plasma [2]. This indicates the possibility that Ti metal and its alloys could form a bone-like apatite layer on their surfaces in the living body and could bond to living bone through the apatite layer, if their surfaces were modified. Apatite formation on a titania gel may be induced by Ti–OH groups that are abundant on the surface of the gel. Such Ti–OH groups could be formed on the surfaces of Ti metal and its alloys in the living body, if, for example, their surfaces are modified with a sodium titanate layer and its Na^+^ ions are exchanged with H_3_O^+^ ions in a body fluid. Such a sodium titanate layer could be formed on these surfaces by a chemical treatment.

In this paper, various types of bioactive titanate layers formed on the surfaces of Ti metal and its alloys using chemical treatments, as well as their clinical applications, are reviewed.

## 2. Bioactive Sodium Titanate

**Figure 1:**
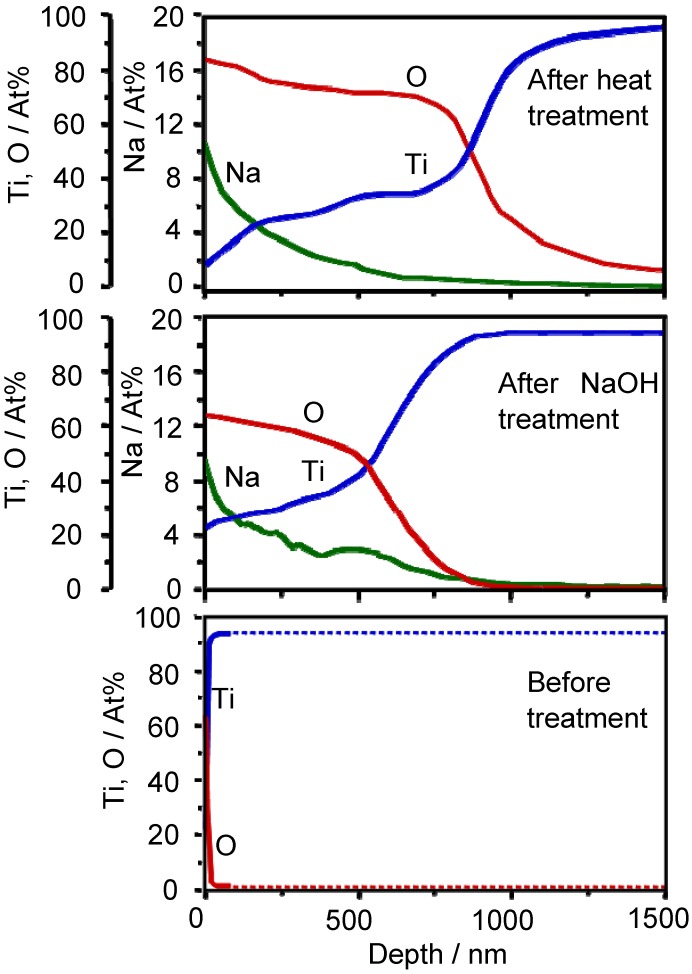
AES depth profiles of the surfaces of Ti metals untreated and those subjected to NaOH and heat treatments.

When Ti metal is soaked in a 5 M NaOH aqueous solution at 60 °C for a period of 24 h, sodium and oxygen ions penetrate into the surface of the Ti metal to a depth of 1 μm, and only the oxygen ions penetrate into deeper regions on subsequent heat treatment at 600 °C for a period of 1 h in an ambient atmosphere, as shown by the Auger electron spectra in Figure 1 [3].

**Figure 2:**
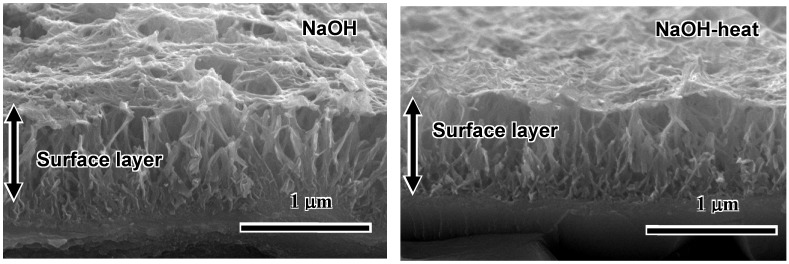
SEM photographs of cross sections of surfaces of Ti metals subjected to NaOH and heat treatments.

A fine network structure on the nanometre scale, consisting of feather-like phases, is formed on the surface of Ti metal with a thickness of 1 μm by the NaOH treatment, and this densifies after the subsequent heat treatment, as shown by the SEM photographs in Figure 2 [4].

**Figure 3:**
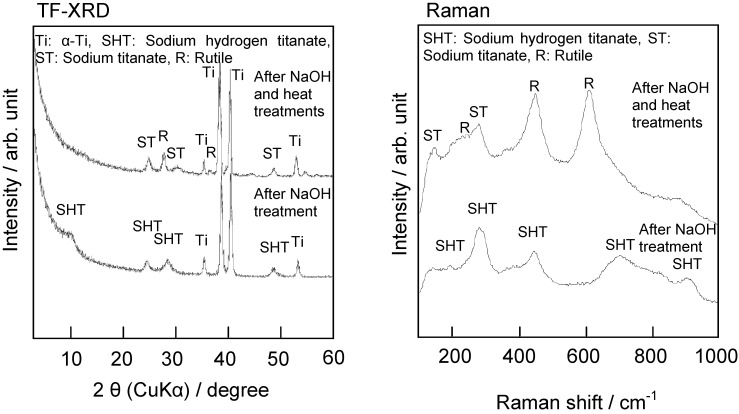
TF-XRD patterns and Raman spectra of surfaces of Ti metals subjected to NaOH and heat treatments.

**Figure 4:**
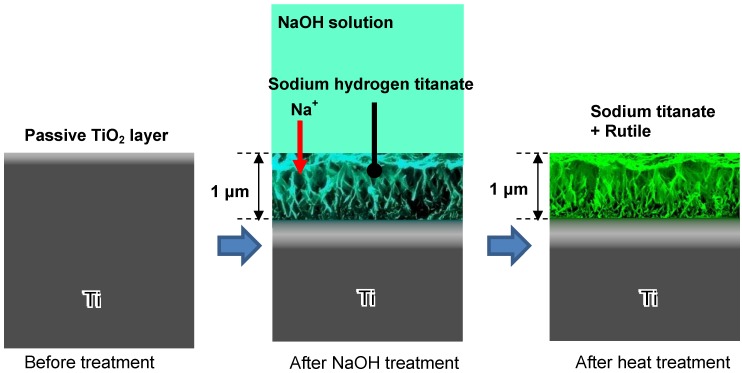
Structural change of surface of Ti metal subjected to NaOH and heat treatments.

The fine network structure formed by the NaOH treatment is composed of nano-sized sodium hydrogen titanate (Na*_x_*H*_2-x_*Ti*_y_*O*_2y+1_*, where 0 < *x* < 2 and *y* = 2, 3, or 4), and this is converted into sodium titanate (Na_2_Ti*_y_*O*_2y+1_*, *y* = 5, 6…) and rutile by the subsequent heat treatment, as shown by thin-film X-ray diffraction pattern and Raman spectra in Figure 3 [5]. These structural changes due to the NaOH and heat treatments are shown schematically in Figure 4.

The scratch resistance of this surface layer as formed by the NaOH treatment is poor, but it increases markedly on subsequent heat treatment, from 5 to 50 mN [5].

**Figure 5:**
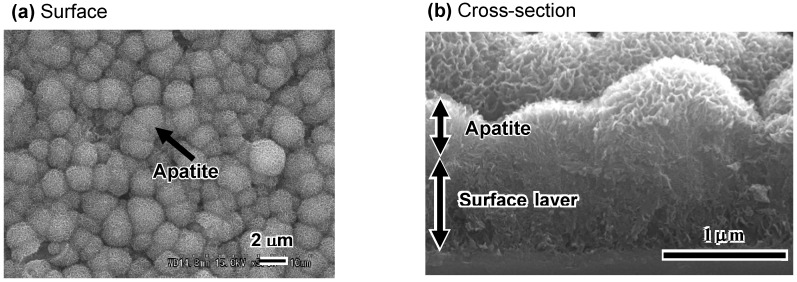
SEM photographs of surface (a) and cross-section (b) of NaOH- and heat-treated Ti metal after soaking in SBF for 1 d.

**Figure 6:**
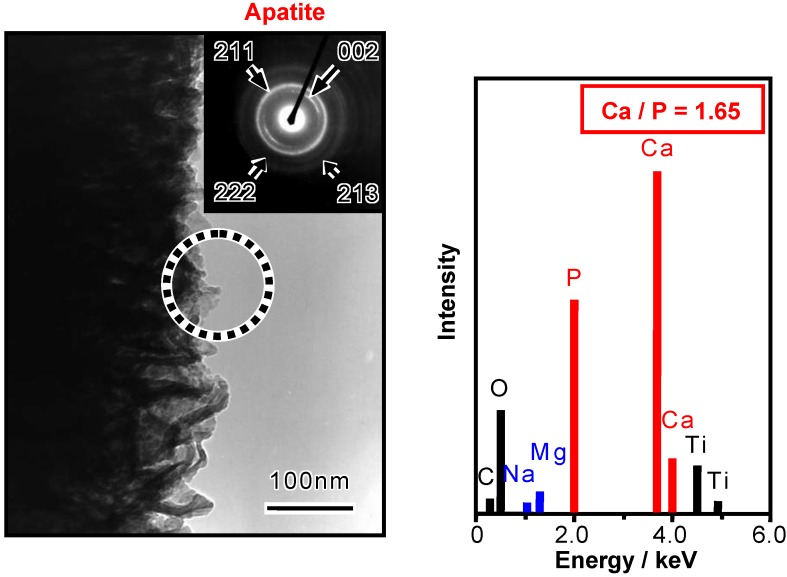
TEM photograph and EDX spectrum of the surface of the NaOH- and heat-treated Ti metal after soaking in SBF for 5 d (dotted circle: area of electron diffraction and EDX analysis).

When the thus-treated Ti metal is soaked in SBF, bone-like apatite begins to precipitate in the deep regions of the interspaces of the feather-like phases, and grows and fills these interspaces to integrate with the feather-like phases, forming a dense composite of apatite and titanate, which eventually grows over the surface layer, as shown in the SEM photographs in Figure 5(a) and Figure 5(b) [4]. The thus-formed apatite is confirmed to take a needle-like form, and has a composition with a Ca/P ratio = 1.65, accompanied with small amounts of Na and Mg, similar to the bone mineral, from the transmission electron micrograph and energy dispersive X-ray analysis (EDX) shown in Figure 6 [6].

**Figure 7:**
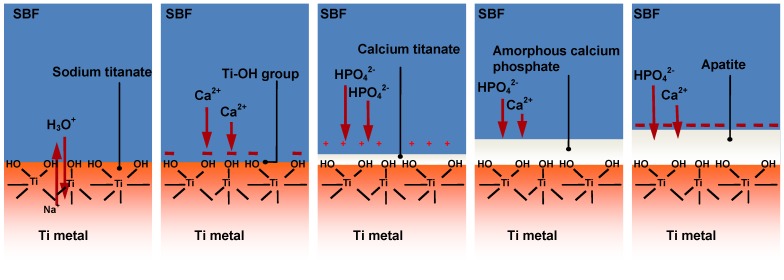
Process of apatite formation on NaOH- and heat-treated Ti metal in SBF.

According to X-ray photoemission spectroscopic (XPS) analysis [7], transmission electron microscopic (TEM) observation [6] and zeta potential measurement [8] of the surfaces of treated Ti metal as a function of soaking time in SBF, bone-like apatite is formed by the following process on the Ti metal in SBF, as shown in Figure 7. Sodium ions in the sodium titanate at the surface of the Ti metal are exchanged with the H_3_O^+^ ions in SBF. As a result, Ti–OH groups are formed on the surface of the Ti metal and the pH of the surrounding SBF increases from the released Na^+^ ions. In such high a pH environment, the Ti–OH groups are negatively charged [9] and preferentially combine with the positively charged Ca^2+^ ions in SBF. As the calcium ions accumulate, the surface becomes positively charged and combines with negatively charged phosphate ions to form an amorphous calcium phosphate. This calcium phosphate eventually transforms into a stable crystalline bone-like apatite [8].

**Figure 8:**
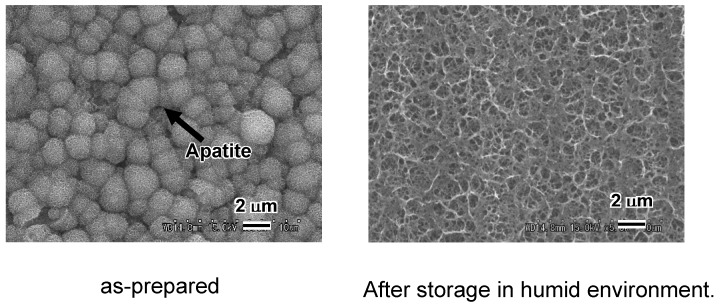
SEM photographs of surfaces of Ti metals soaked in SBF for 1 d, just after NaOH and heat treatments, and after subsequent storage in 95% relative humidity at 80 °C for 1 week.

The Na^+^ ions in the sodium titanate on the surface of Ti metal are easily released, even when the Ti metal is stored in a humid environment. This is a problem in clinical applications, since any decrease in the sodium concentration of the sodium titanate decreases the apatite-forming ability of the treated Ti metal in a body environment, as shown in Figure 8 [5].

This problem can be resolved by the controlled release of Na^+^ ions from the top surface of the treated Ti metal before the heat treatment stage. For example, when Ti metal is soaked in water for a period of 3 h after the NaOH treatment, one-third of the sodium ions of the surface sodium hydrogen titanate layer are released from the top surface via exchange with the H_3_O^+^ ions in the water. The thus-treated Ti metal then forms anatase on the top surface after a heat treatment. The thus-formed anatase is stable in a humid environment and has a high apatite-forming ability in a body environment where the pH is increased by the sodium ions released from the inner layer. As a result, the thus-treated Ti metal exhibits a high apatite-forming ability, and is stable in a humid environment [5].

**Figure 9:**
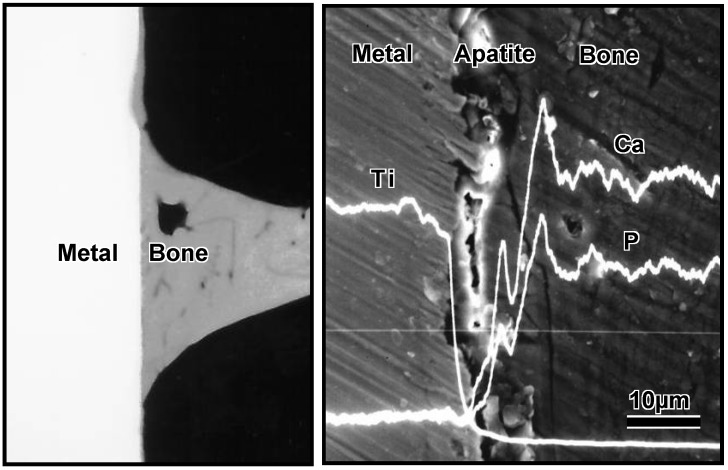
CMR and SEM-EDX photographs at the interface between Ti metal which was subjected to NaOH and heat treatments, and implanted into rabbit tibial bone for eight weeks.

**Figure 10:**
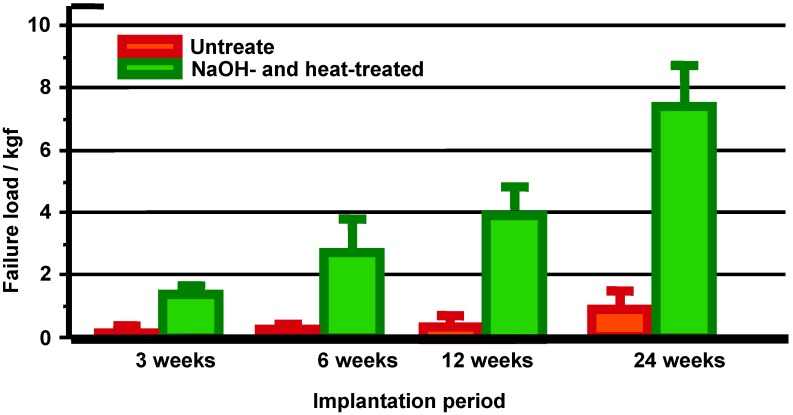
Detaching failure loads of the untreated and the NaOH- and heat-treated Ti metals as a function of implantation period in rabbit tibia.

These *in vitro* results indicate that treated Ti metal forms bone-like apatite on its surface in bone defects, and bonds to the surrounding bone through this apatite layer. As expected, it formed apatite on its surface when it was implanted into the tibia of a rabbit and came into direct contact with the surrounding bone through the apatite layer without intervention of the fibrous tissue, as shown in Figure 9 [10]. When a tensile stress was applied to the interface, a higher load was required to produce fractures at the interface for the treated Ti metal than for the untreated Ti metal, and the difference between these samples increased with increasing period after implantation, as shown in Figure 10.

**Figure 11:**
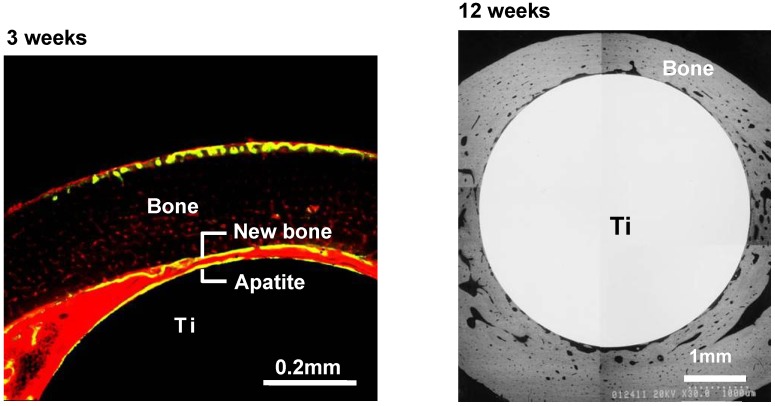
Confocal laser scanning microscopy photograph (left-hand side) and SEM photograph (right-hand side) of the cross-section of the NaOH- and heat-treated Ti metal implanted into rabbit femur for three and 12 weeks, respectively.

**Figure 12:**
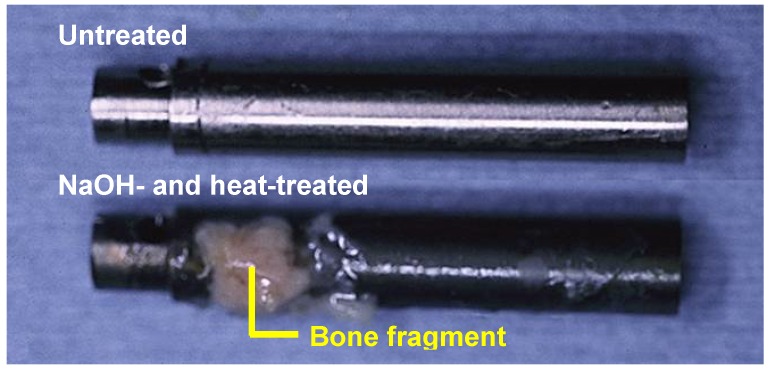
Surfaces of the untreated and the NaOH- and heat-treated Ti implants after the pull-out tests (12 weeks after implantation).

**Figure 13:**
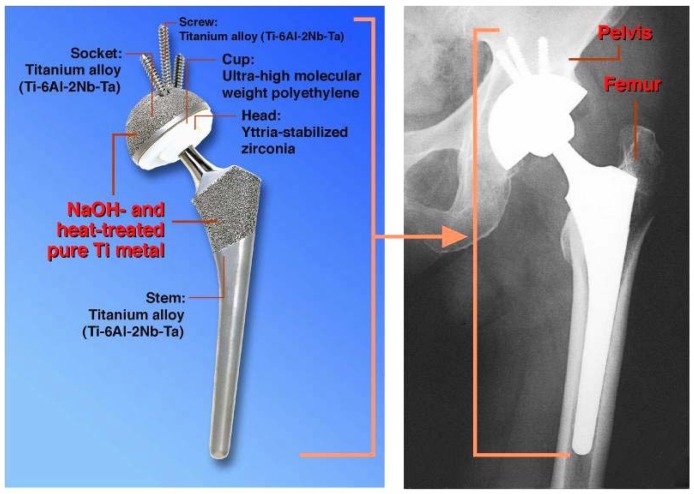
Clinical application of NaOH- and heat-treated Ti metal in hip joint system.

When similarly treated Ti metal rods were implanted into the medullary canal of a rabbit’s femur, they also formed apatite on their surfaces, and were soon fully surrounded by newly grown bone, as shown in Figure 11. These could not be pulled out from the surrounding bone without being accompanied by bone fragments, as shown in Figure 12 [11]. Based on these *in vitro* and *in vivo* results, the NaOH and heat treatments were applied to a porous Ti metal layer formed on the Ti-6Al-2Nb-Ta alloy of a total hip joint. The thus-prepared bioactive hip joint has been clinically used in Japan since 2007, as shown Figure 13 [12].

## 3. Bioactive Calcium Titanate

The NaOH and heat treatments described above are also effective for Ti-based alloys, such as Ti-6Al-4V, Ti-6Al-2Nb-Ta, and Ti-15Mo-5Zr-3Al in inducing bioactivity [13,14], but not for new types of Ti-Zr-Nb-Ta alloys that are free from cytotoxic elements.

**Figure 14:**
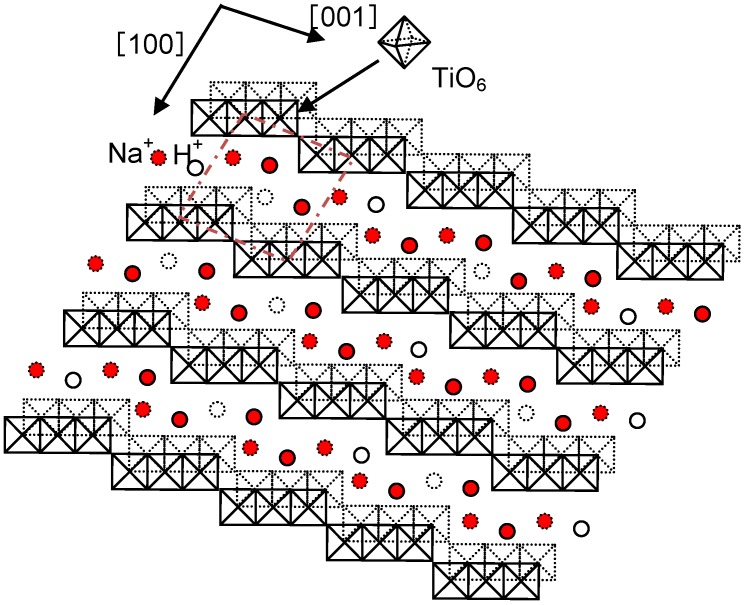
Structure of sodium hydrogen titantate [15].

The sodium hydrogen titanate formed on Ti metal and its alloys by the NaOH treatment has a layered structure, as shown in Figure 14 [15]. The sodium ions in the interlayers are speculated to be easily replaced by the calcium ions in a calcium solution. Ti metal and its alloys treated using the NaOH solution are expected to form a calcium hydrogen titanate layer after the calcium solution treatment, and to form calcium titanate after a subsequent heat treatment. The resulting product could exhibit a high apatite-forming ability, as well as a higher stability in a humid environment, since the calcium ions released from the surface layer could increase the ionic activity product of the apatite in the surrounding fluid more effectively than sodium ions can, and the diffusion coefficient of the calcium ions in the surface layer would be lower than that of sodium ions.

**Figure 15:**
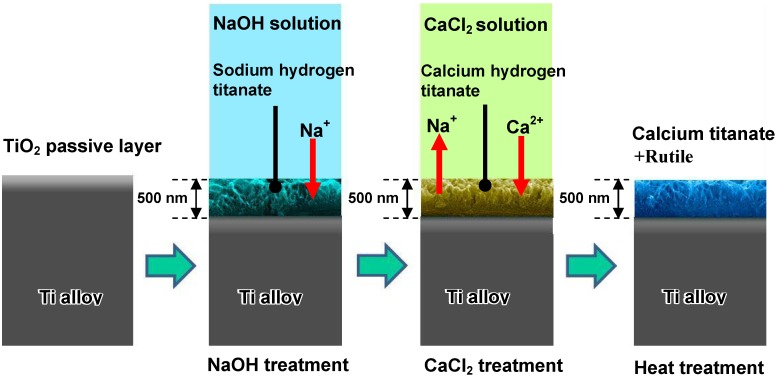
Structural changes of surface of Ti-15Zr-4Nb-4Ta alloy subjected by NaOH, CaCl_2_ and heat treatments.

When Ti-15Zr-4Nb-4Ta alloy was soaked in 100 mM of a CaCl_2_ solution at 40 °C for a period of 24 h after the NaOH treatment, the sodium hydrogen titanate formed on the alloy surface by the NaOH treatment was isomorphously transformed into calcium hydrogen titanate. This phase was converted into calcium titanate and rutile by a subsequent heat treatment at 600 °C for a period of 1 h, as shown in Figure 15 [16]. Unfortunately, the resulting product did not form apatite on its surface in SBF within a period of 3 days. This may be attributable to the extremely low diffusion coefficient of the calcium ions in the calcium titanate.

**Figure 16:**
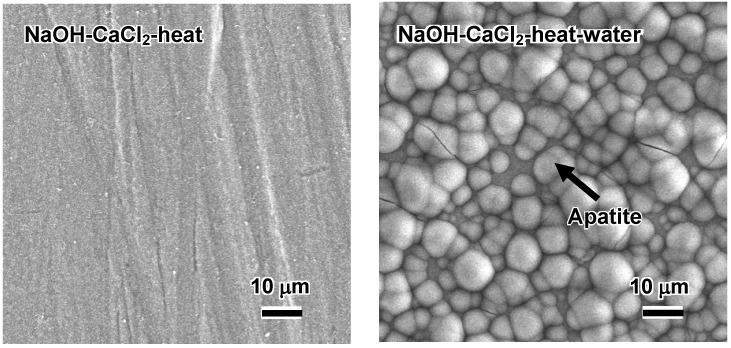
SEM photographs of the surfaces of Ti-15Zr-4Nb-4Ta alloy soaked in SBF for 3 d after NaOH, CaCl_2_ and heat treatments, and subsequent water treatment.

**Figure 17:**
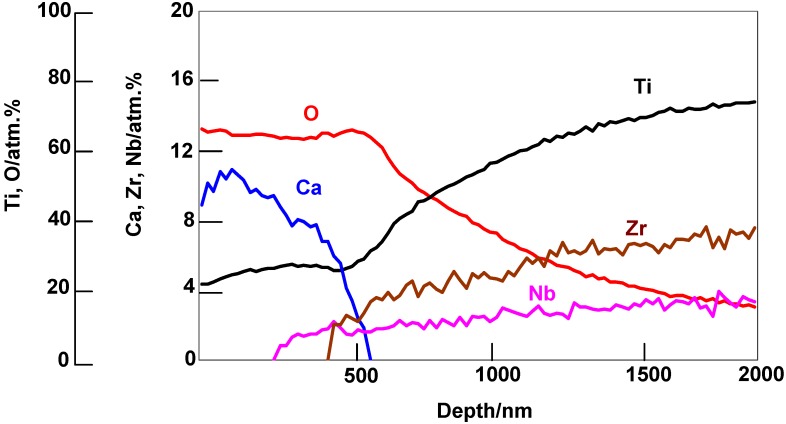
AES depth profiles of the surface of Ti-15Zr-4Nb-4Ta alloy subjected to NaOH, CaCl_2_, heat and water treatments.

Such an alloy did form apatite on its surface in SBF within 3 days after it was soaked in water at 80 °C for a period of 24 h, as shown in Figure 16 [16]. This is attributed to an increase in diffusion coefficient of the calcium ions in the calcium titanate by the partial replacement of the calcium ions with H_3_O^+^ ions during the hot-water treatment. This partial replacement was confirmed by the depth profile of the Auger electron spectra of the resulting product shown in Figure 17 [16]. A decrease in Ca concentration near to the top surface is due to the ion exchange of Ca^2+^ ions with H_3_O^+^ ions.

**Figure 18:**
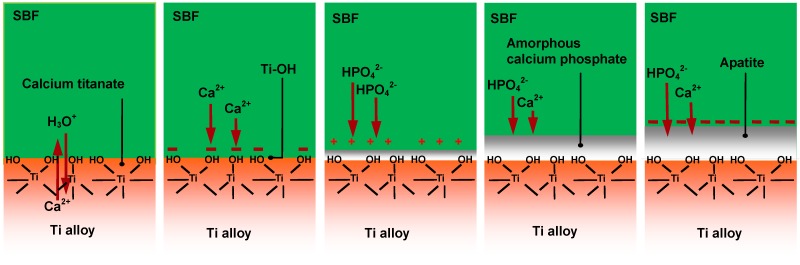
Process of apatite formation on Ti-15Zr-4Nb-4Ta alloy subjected to NaOH, CaCl_2_, heat and water treatments in SBF.

Apatite formation on an alloy enriched with calcium ions on its surface in SBF is considered to proceed by the process shown in Figure 18. The calcium ions are released from the surface via exchange with H_3_O^+^ ion in SBF to produce Ti–OH groups on the surface. The thus-formed Ti–OH groups combine with the calcium ions. As the calcium ions accumulate, the surface becomes positively charged and combines with negatively charged phosphate ions forming calcium phosphate, which eventually transforms into apatite.

The thus-treated alloy does not show any decrease in its apatite-forming ability in SBF, even when it is stored in a 95% relative humidity at 80 °C for a period of 1 week, as expected [16]. These treatments are also effective in inducing an apatite-forming ability in different types of Ti-Zr-Nb-Ta alloys, such as Ti-29Nb-13Ta-4.6Zr and Ti-36Nb-2Ta-3Zr-0.3O, as well as pure Ti metal.

## 4. Bioactive Titanium Oxide

The sodium hydrogen titanate formed on Ti metal by the NaOH treatment may substitute its Na^+^ ions completely with H_3_O^+^ ions to form hydrogen titanate when it is exposed to water or an HCl solution, and would form titanium oxide by the subsequent heat treatment. The resulting titanium oxide would be expected to exhibit bioactivity, since even a type of pure titania gel forms bone-like apatite on its surface in SBF [17]. If bioactive titanium oxide is obtained by such chemical and thermal treatments, then such treatments can be applied to porous titanium metals for forming a uniform bioactive layer on walls of the pores, even in deep regions. The thus-formed bioactive layer does not disturb the biological environment by the released ions, even of the narrow spaces of the pores.

**Figure 19:**
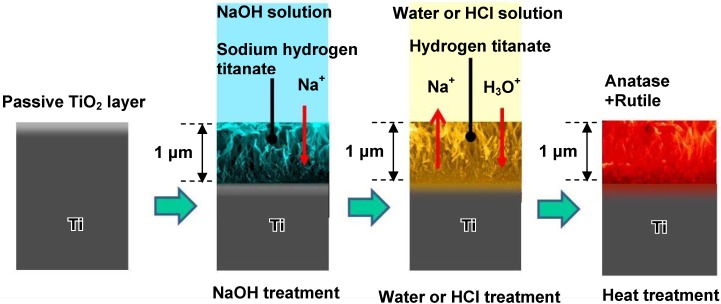
Surface structural change of Ti metal by NaOH, water or HCl and heat treatments.

When Ti metal was soaked in pure water or an HCl solution with different concentrations ranging from 0.5 to 100 mM at 40 °C for a period of 24 h after the NaOH treatment, the sodium hydrogen titanate formed on the Ti metal by the NaOH treatment was isomorphously transformed into hydrogen titanate. This phase was converted into anatase and/or rutile TiO_2_ by a subsequent heat treatment at 600 °C for a period of 1 h, as shown in Figure 19 [18].

**Figure 20:**
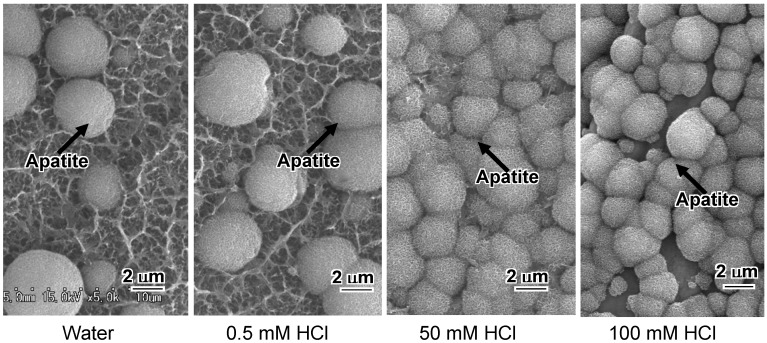
SEM photographs of surfaces of Ti metals soaked in SBF for 1 d, after NaOH, water or HCl and heat treatments.

The heat-treated Ti metal formed apatite on its surface in SBF within 1 day, as shown in Figure 20. Its apatite-forming ability increased with increasing concentration of the HCl solution. Its apatite-forming ability did not depend upon the anatase to rutile ratio. The high apatite-forming ability of Ti metal treated with high concentration HCl solutions can be attributed to the high positive surface charge of the titanium oxide formed on it. Titanium oxide is usually positively charged in acidic solutions [9]. Positively charged surface can induce apatite deposition by preferential adsorption of negatively charged phosphate ions in SBF, in contrast with the preferential adsorption of the positively charged calcium ions on the negatively charged surface of Ti metal subjected to NaOH and heat treatments.

**Figure 21:**
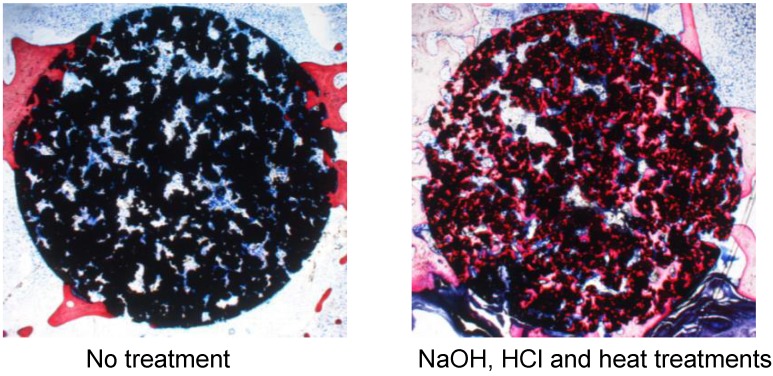
Bone formation in porous Ti metals subjected to no treatment and NaOH, HCl and heat treatments, 26 weeks after implantation into rabbit femur.

**Figure 22:**
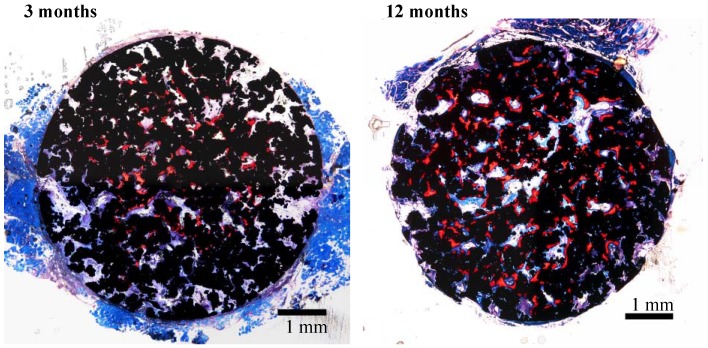
Bone formation in porous Ti metal subjected to NaOH, HCl and heat treatments, 3 and 12 months after implantation into muscle of beagle dog. Stain: Stevenel’s blue and Van Gieson’s picrofuchsin. Reprinted from Ref. [20] with permission from Elsevier.

**Figure 23:**
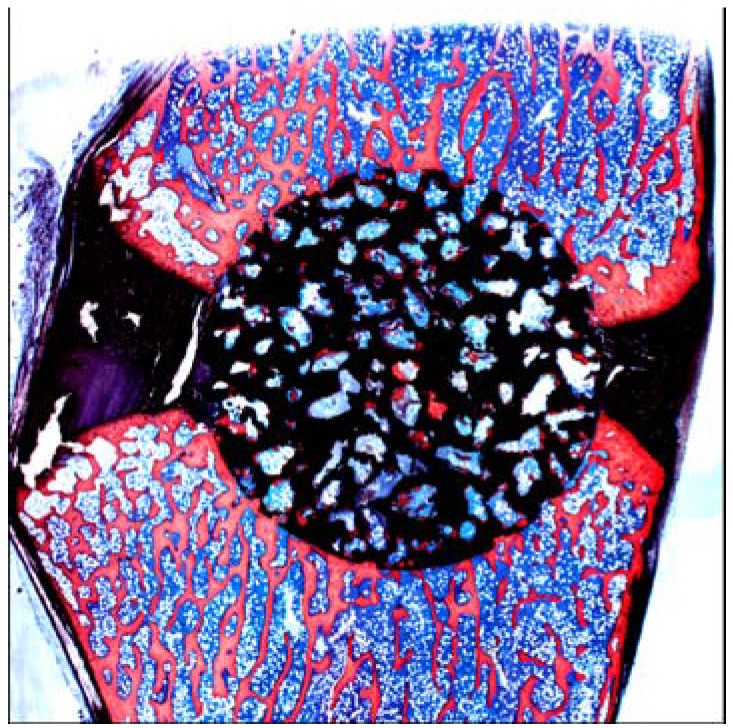
Fusion of spine of dog by a bioactive porous Ti metal. Reprinted from Ref. [21] with permission from American Association of Neurological Surgeons.

A porous Ti metal sample containing 50 to 70 vol % of interconnected pores 200–500 μm in diameter was subjected to NaOH, 0.5 mM HCl, and heat treatments to form titanium oxide on its pore walls. The thus-treated porous Ti metal was fully penetrated with newly grown bone from its periphery when it was implanted into the femur of a rabbit, as shown in Figure 21 [19]. This porous Ti metal formed bone tissue from its centre when it was implanted into the muscle of a dog, as shown in Figure 22 [20]. These results indicate that the thus-treated porous Ti metal exhibits osteoinductivity, as well as osteoconductivity. This was successfully applied to a spinal fusion device of a dog, as shown in Figure 23 [21], and is subject to clinical tests for a human device.

## 5. Summary

(1) Sodium titanate forms on the surface of Ti metal by NaOH and heat treatments. It forms bone-like apatite on its surface in a body environment and bonds to living bone through the apatite layer. The long-term stability of its apatite-forming ability in a humid environment is improved by removing the Na^+^ ions from its top surface. These treatments were applied to porous Ti metal on a total artificial hip joint of a Ti-6Al-2Nb-Ta alloy. The thus-prepared bioactive hip joint has been clinically used in Japan since 2007.
(2) These treatments are also effective in inducing apatite-forming ability in conventional Ti-based alloys, such as Ti-6Al-4V, Ti-6Al-2Nb-Ta, and Ti-15Mo-5Zr-3Al, but not for new types of Ti-Zr-Nb-Ta alloys, such as Ti-15Zr-4Nb-4Ta, Ti-29Nb-13Ta-4.6Zr, and Ti-36Nb-3Zr-0.3O that are free from cytotoxic elements. Calcium titanate forms on the surface of Ti-Zr-Nb-Ta alloys by NaOH, CaCl_2_, and heat treatments. The thus-prepared calcium titanate forms bone-like apatite on its surface in SBF after a subsequent water treatment.
(3) Pure titanium oxide is formed on the surface of Ti metal after NaOH, HCl, and heat treatments. Porous Ti metal subjected to these treatments exhibits osteoinductivity as well as osteoconductivity. This is now under clinical tests for application to a spinal fusion device.
(4) The process of apatite formation on the surfaces of Ti metals and its alloys modified with the different types of titanate layers in a body environment has been discussed in terms of their surface charges.
